# Trends in surgical and ablative treatment of localised renal cell carcinoma: A review of publication trends over 16 years (2000–2015)

**DOI:** 10.1080/2090598X.2019.1590516

**Published:** 2019-05-15

**Authors:** Amelia Pietropaolo, Patrick Jones, Omar M. Aboumarzouk, Bhavan P. Rai, C. Richard W. Lockyer, Matt C. Hayes, Rob Geraghty, Adityanarayan Bhatnagar, Bhaskar K. Somani

**Affiliations:** a Department of Urology, University Hospital Southampton NHS Trust, Southampton, UK; b Department of Urology, Queen Elizabeth University Hospital, Glasgow, UK; c Department of Urology, South Tees Hospitals NHS Foundation Trust, Middlesbrough, UK; d Department of Clinical Oncology, University Hospital Southampton NHS Trust, Southampton, UK

**Keywords:** Publication trends, renal cancer, partial nephrectomy, robotic, cryoablation, radiofrequency ablation

## Abstract

**Objective**: To investigate the bibliometric (publication) trends in surgical and ablative treatment of localised renal cell carcinoma (RCC) over a period of 16-years, from 2000 to 2015, as publication trends reflect clinical practice and new innovations.

**Material and methods**: We performed a systematic review using PubMed over a 16-year period from 2000 to 2015 for all published papers on surgical and ablative management of renal tumours. Data were further analysed in two time periods, period-1 (2000–2007) and period-2 (2008–2015).

**Results**: During the last 16 years a total of 2415 papers were published on surgical (*n* = 1662, 69%) and ablative (*n* = 753, 31%) management of RCC. This included partial nephrectomy (PN; *n* = 1662, 69%), cryoablation (CA; *n* = 405, 17%), and radiofrequency ablation (RFA; *n* = 348, 14%). When comparing the two time periods for PN, during period-2, the change was +189% (*P* < 0.001), +69% (*P* = 0.004) and +4600% (*P* < 0.001) for open PN, laparoscopic PN and robotic PN, respectively. Regarding ablative techniques, a change of +109% (*P* = 0.002) and +78% (*P* = 0.036) was seen for CA and RFA, respectively. There was also a significant rise in percutaneous CA when compared to laparoscopic CA (*P* < 0.002).

**Conclusions**: There has been a rise in all forms of PN and ablative techniques over the last 16 years. This rise has been particularly steep for robotic PN potentially reflecting a change in surgical practice.

**Abbreviations**: CA: cryoablation; CC: correlation coefficient; MIS: minimally invasive surgery/surgical; NSS: nephron-sparing surgery; (L)(O)(R)PN: (laparoscopic) (open) (robotic) partial nephrectomy; PRISMA: Preferred Reporting Items for Systematic Reviews and Meta-Analyses; RFA: radiofrequency ablation; RN: radical nephrectomy; SRM: small renal mass

## Introduction

RCC represents 3.5% of all malignancies and over the last two decades the incidence of RCC has increased by ~2% [1]. This has been fuelled by an increase in incidentally diagnosed tumours on radiological imaging such as ultrasonography and CT. Incidental renal tumours now account for 48–66% of all renal tumours [2,3]. While these help in early diagnosis of tumours, they are usually localised RCC allowing for nephron-sparing surgery (NSS) and ablative treatments [1,4].

Treatment of localised RCC has shifted from radical nephrectomy (RN) to NSS and ablative therapies such as cryoablation (CA) and radiofrequency ablation (RFA) [5,6]. Minimally invasive surgical (MIS) techniques allow for preservation of renal function with oncological outcomes similar to RN [1]. The selection of a specific treatment option should be based on a patient’s health and comorbidities, oncological potential of the tumour, and effectiveness/morbidity associated with the type of treatment offered, with patient counselling and shared decision-making paramount to all these [7].

Currently no recommendation is made for small renal masses (SRMs), and patient preference and available surgical expertise seem to guide the treatment offered. With a rising burden of SRMs, health commissioners and clinicians need to justify resource allocation on treatment of SRMs with manpower and personnel allocated to the job. Publication trends reflect changes in trends of clinical interventions and it is therefore likely that these areas perceived to be more important attract more funding [8]. Although there seems to be a wider uptake of partial nephrectomy (PN) and ablative techniques, there is no bibliometric study that addresses the publication trends on it. We therefore conducted the present study to analyse the publication trends (PubMed) associated with surgical/ablative management of renal masses, including PN, CA and RFA over the last 16 years (2000–2015).

## Materials and methods

We conducted a systematic review of the literature using Medical Subject Headings (MeSH) terms, title words, and keywords in PubMed/MEDLINE over the last 16 years, from January 2000 to December 2015, for all published papers on ‘surgical/ablative management of renal tumours’.

### Evidence acquisition: Criteria for including studies for this review

*Inclusion criteria*:

- All English language studies.
- All non-English studies with abstracts written in the English language.
- Studies reporting on surgical and ablative treatment for localised renal cancer – partial nephrectomy (PN), open surgery, laparoscopic surgery, robotic surgery, radiofrequency ablation (RFA), and cryoablation (CA).

*Exclusion criteria*:

- Benign renal tumours.
- Urothelial tumours and treatment associated with them.
- Studies without a published abstract.
- Animal and laboratory studies.
- Case reports.

### Search strategy and study selection

The systematic review was performed according to the Cochrane Review and the Preferred Reporting Items for Systematic Reviews and Meta-Analyses (PRISMA) guidelines. The search strategy was conducted for all relevant abstracts regarding each specific intervention, which was analysed year by year from 2000 to 2015 (16 years) on PubMed. Specific terminology used was different for each topic. We examined all published papers on ‘renal cancer’, ‘treatment’, ‘nephrectomy’, ‘open nephrectomy’, ‘localised renal tumour’, ‘small renal mass’, ‘partial nephrectomy’, ‘robotic assisted nephrectomy’, ‘laparoscopic nephrectomy’, ‘robotic partial nephrectomy’, ‘laparoscopic partial nephrectomy’, ‘percutaneous ablation’, ‘cryoablation’, ‘radiofrequency ablation’, ‘RFA’, ‘surgical treatment’ and ‘ablative treatment’.

Boolean operators (AND, OR) were used to refine the search. There were no language restrictions and all non-English language papers with published English abstracts were also included in our review, added to the total and subsequently analysed in a separate subgroup. Whilst review articles were included, case reports and studies without a published abstract were excluded. For the purposes of this study, we included laparoscopic PN (LPN), open PN (OPN), robotic PN (RPN), CA and RFA (both laparoscopic and percutaneous approach) in the surgical/ablative management of renal tumours.

To make an effective comparison and to identify, compare and contrast the main different features, the data derived from each single research have been divided into two 8-year periods, period-1 (2000–2007) and period-2 (2008–2015). Data were collected using Microsoft Excel 2016 (version 16.0) and analysed using the independent *t-*test and Pearson’s correlation coefficient, using the Statistical Package for the Social Sciences (SPSS®) version 24 (SPSS Inc., IBM Corp., Armonk, NY, USA).

## Results

**Figure 1.**
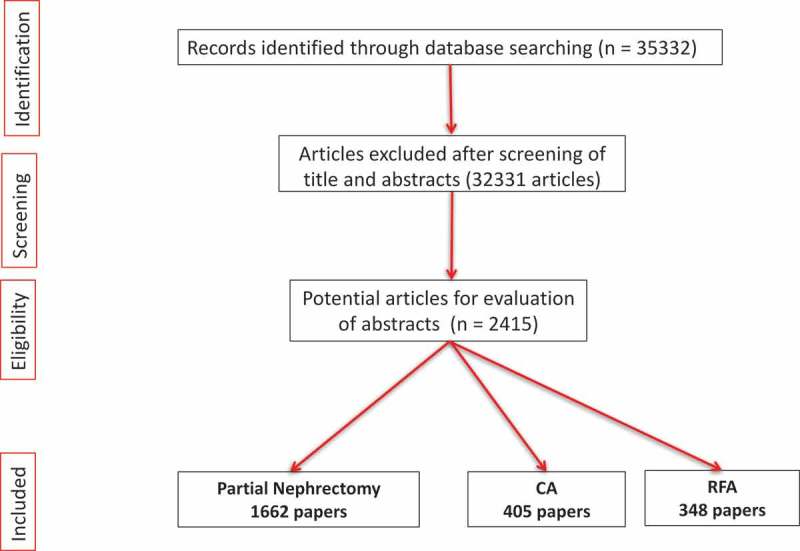
PRISMA flowchart.

During the last 16 years, a total of 2415 papers were published on ‘surgical/ablative management of renal tumours’ (Figure 1).

### PN

**Figure 2.**
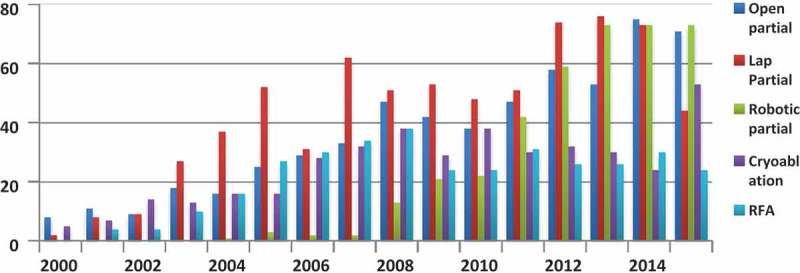
Breakdown of trends by year.

**Figure 3.**
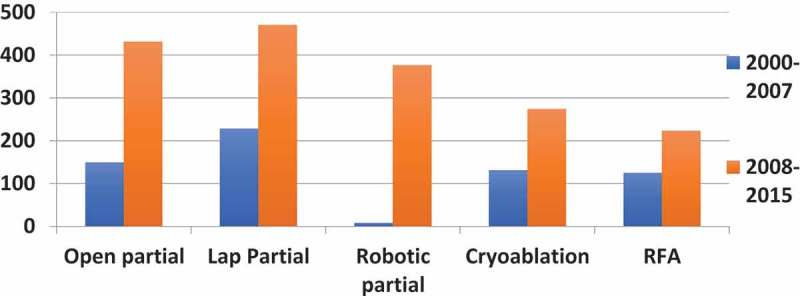
Trends of PN and ablative strategies.

PN has replaced RN as the treatment option for SRMs, with a total of 1662 (69%) papers published over the last 16 years. There was a significant positive correlation in publications on OPN (*P* < 0.001, correlation coefficient [CC] = 0.973), LPN (*P* < 0.001, CC = 0.758) and RPN techniques (*P* < 0.001, CC = 0.980) of PNs (linear trend) over this time period. The overall number of papers rose significantly from 385 papers in period-1 to 1277 papers in period-2, a rise of 232% (*P* < 0.001, 95% CI 69.45–153.55) (Figures 2 and 3).

When comparing the two time periods, OPN increased by 189% from 149 (period-1) to 431 (period-2) (*P* < 0.001, 95% CI 22.86–47.65); LPN increased by 69% from 278 (period-1) to 470 (period-2) (*P* = 0.004, 95% CI 11.01–49.43) and RPN increased by 4600% from eight (period-1) to 376 (period-2) (*P* < 0.001, 95% CI 24.44–67.56) (Figure 3).

### Ablative techniques

A total of 753 (31%) papers were published on ablative techniques over the 16-year period (405 papers on CA and 348 papers on RFA) (Figures 2 and 3). There was an overall increase of 94% in period-2, with published papers on CA and RFA rising by 109% and 78%, respectively. While CA increased from 131 (period-1) to 274 (period-2) (*P* = 0.002, 95% CI 8.08–27.67), RFA rose from 125 (period-1) to 223 (period-2) (*P* = 0.036, 95% CI 0.97–23.53).

Looking at the techniques of CA and RFA, 371 papers clearly mentioned whether they used a percutaneous or laparoscopic approach to their ablative technique. Of those reporting on CA (*n* = 174), 110 (63%) reported a percutaneous approach and 64 (37%) reported on a laparoscopic approach. Whilst a percutaneous approach increased by 143% in the second time period (from 32 in period-1 to 78 in period-2), the laparoscopic approach remained exactly the same in the two time periods (32 each in both periods). Of the studies reporting on RFA (*n* = 197), 173 (88%) reported a percutaneous approach and 24 (12%) reported a laparoscopic approach. Whilst a percutaneous approach increased by 37% in the second time period (from 73 in period-1 to 100 in period-2), the number of papers on a laparoscopic approach still remained very small (nine in period-1 and 15 in period-2). When comparing the two approaches, the rise in percutaneous CA was significantly higher in the second time period compared to laparoscopic CA (*P* < 0.002).

### Comparative studies on different techniques

**Figure 4.**
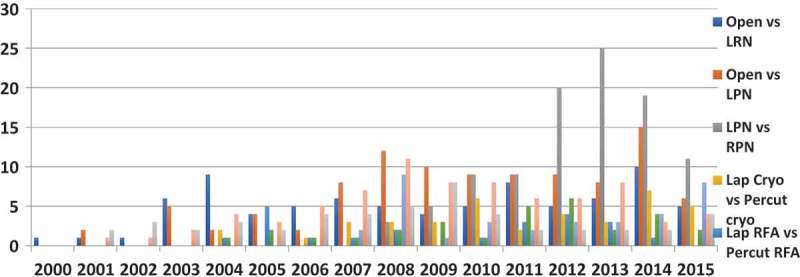
Breakdown of comparative studies by year.

Given the lack of a recommended treatment for SRMs, several comparatives studies have been carried out over the 16-year period showing an overall four-fold increase in publications over the two time periods (Figure 4). During the second time period the majority of comparative studies published were about LPN vs RPN (*n* = 101) with a steep increase in the last few years. Similarly, during period-2 there were 25 studies on CA vs PN, 33 on RFA vs PN, and 54 studies on CA vs RFA.

Approaches to ablative interventions over the 16-year period included comparative studies of percutaneous and laparoscopic RFA (22 studies), percutaneous and laparoscopic CA (39 studies) and CA vs RFA vs PN (49 studies) (Figure 4).

## Discussion

This is one of the first bibliometric studies in the field of RCC looking at publication trends of ‘surgical/ablative treatment for localised renal cancer’ over the last 16 years (2000–2015). Overall, the number of papers published on surgical and ablative treatments has trebled over the second time period (641 in period-1 to 1774 in period-2).

### Meaning of the study

Publication trends would indicate that PN and ablative treatments have both increased. PN has increased more than three-fold (×3.3) in the second time period reflecting a growth of OPN (×2.9), LPN (×2.1) and RPN (×47). All approaches of PN saw a significant rise in the second time period but this was most evident for RPN.

For ablative techniques, compared to the laparoscopic approach, publications on percutaneous CA have increased over the second time period (×2.4). Similarly, there were more published papers on CA (×1.3) than RFA in the second time period. Percutaneous CA seems to be a significantly more published technique than the laparoscopic CA, perhaps reflecting our current clinical practice.

### Practical considerations, reflection on published guidelines and limitations of our study

Publications trends tend to reflect urological practice, recommended guidelines and potentially gives an insight into clinically important interventions in the area of interest. Treatment decisions need to be made on the complexity of the tumours, functional status of the patients, and the effectiveness and morbidity of the intervention, and these should be discussed on an individualised basis [7].

With a rapid increase in the diagnosis of SRMs, and guidelines recommending MIS [1,7], the growth of published articles on PN and ablative treatments was inevitable [9,10]. This correlated with a simultaneous decline in RN reflecting this move towards MIS.

A rapid interest and uptake of laparoscopic and then robotic surgery led to a rise in PN, with RPN now considered safe and offering a superior morbidity profile compared to LPN [10,11]. Similarly, a trend of percutaneous approach with ablative techniques seems to have become more popular compared to their laparoscopic counterparts, with percutaneous CA possibly gaining the most popularity in recent times, although the results of CA seem to be broadly similar to RFA [12]. Ablative techniques help to avoid extirpative surgery with shorter hospital stay and faster recovery [13].

With increasing burden on healthcare resources, financial expenditure on research and treatment related to SRMs needs to be justified. Publication trends might help justify healthcare resource allocation, and interventions that are more quality and cost justifiable and efficient are likely to attract more funding.

In our present review, we did not cover surveillance or other non-surgical forms of treatment for RCC, nor did we quantify advanced or metastatic disease or publications reporting on RN. Although published data gives insight into interventions, newer minimally invasive treatments are more likely to get published due to the novelty attached to them, potentially having a publication bias attached to them. We did not evaluate the studies based on systems like the PADUA (Preoperative Aspects and Dimensions Used for an Anatomical) and R.E.N.A.L. (Radius; Exophytic/Endophytic; Nearness; Anterior/Posterior; Location) nephrometry score systems, which might have added more information on the nature of these tumours.

Our present review also showed a number of comparative studies, reflecting a renewed interest in identifying the ideal treatment for SRMs. This not only helps to broaden the surgeons’ therapeutic armamentarium, but also to customise the approach to each specific patient. The rates of PN (all approaches) seem to overwhelmingly dominate the publication trends throughout the entire period, confirming its effectiveness and further establishing its role as the ‘gold standard’ in the treatment of SRMs.

### Strengths and weakness of bibliometric trend analysis

We used PubMed for bibliometric analysis of trends on ‘surgical and ablative management of renal cancer tumours’ over the last 16 years, as it gives a more realistic view of research trends in the field of oncology [14]. The results reflect a global and extensive rise in the publications in some of the fields analysed. Although PubMed is an excellent source for bibliometric analysis compared to Scopus or Web of Science, occasionally there might be journals that are not indexed on PubMed [15]. Although with certain limitations, as most bibliometric analysis are biased toward English language articles, we wanted to make it more comprehensive and therefore also included all non-English language articles that had a published abstract on PubMed.

Although our present review reflects publication trends of treatment associated with localised RCC, this may not reflect the individual interventions offered by clinicians/hospitals. Unless a centre or institution can offer all types of treatments, it is very difficult to achieve equipoise for patient counselling and shared decision-making. Future studies that analyse the citation index and uses multiple databases might be more comprehensive and provide more detailed information. This might also help in identifying landmark papers in renal cancer management and articles that are most cited in the literature. A sharp rise in RPN reflects its non-existence two decades ago and whilst its uptake has increased over the last decade, this disproportionately large increase in the robotic approach is also an inherent disadvantage of bibliographic study.

## Conclusions

Published papers on surgical and ablative treatment for RCC have increased over the last 16 years. Although there has been a rise in publications for all forms of PN, this has been most steep for the robotic approach. Similarly although ablative treatments have risen, this increase has been highest for percutaneous CA. These results might help in patient counselling, resource planning, adequate staffing, and the availability of interventions for renal cancer.
